# Morita-Baylis-Hillman Adducts Display Anti-Inflammatory Effects by Modulating Inflammatory Mediator Expression in RAW264.7 Cells

**DOI:** 10.1155/2017/6898505

**Published:** 2017-07-12

**Authors:** Glaucia V. Faheina-Martins, Jacqueline Alves Leite, Bruna Braga Dantas, Cláudio G. Lima-Júnior, Mário L. A. A. Vasconcellos, Sandra Rodrigues-Mascarenhas, Demetrius A. M. Araújo

**Affiliations:** ^1^Laboratório de Biotecnologia Celular e Molecular, Departamento de Biotecnologia, Centro de Biotecnologia, Universidade Federal da Paraíba, Campus I, João Pessoa, PB, Brazil; ^2^Laboratório de Imunofarmacologia, Departamento de Biologia Celular e Molecular, Centro de Biotecnologia, Universidade Federal da Paraíba, Campus I, João Pessoa, PB, Brazil; ^3^Laboratório de Síntese Orgânica Medicinal da Paraíba (LASOM-PB), Departamento de Química, Universidade Federal da Paraíba, Campus I, João Pessoa, PB, Brazil

## Abstract

Inflammatory response plays an important role not only in the normal physiology but also in pathologies such as cancers. The Morita-Baylis-Hillman adducts (MBHA) are a novel group of synthetic molecules that have demonstrated many biological activities against some parasitic cells such as *Plasmodium falciparum*, *Leishmania amazonensis*, and *Leishmania chagasi*, and antimitotic activity against sea urchin embryonic cells was also related. However, little is known about the mechanisms induced by MBHA in inflammatory process and its relation with anticancer activity. The present work investigated the cytotoxicity of three MBHA derivatives (A2CN, A3CN, and A4CN), on human colorectal adenocarcinoma, HT-29 cells, and their anti-inflammatory activities were examined in lipopolysaccharide- (LPS-) stimulated RAW264.7 macrophage cells, being these derivatives potentially cytotoxic to HT-29 cells. Coincubation with A2CN, A3CN, or A4CN and LPS in RAW264.7 cells inhibited NO production, as well as the production of reactive oxygen species (ROS) was also repressed. The mRNA expressions of IL-1*β* and IL-6 were significantly downregulated by such MBHA compounds in RAW264.7 cells, but only A2CN was able to inhibit the COX-2 gene expression. We also showed that MBHA compounds decreased almost to zero the production of IL-1*β* and IL-6. These findings display that such MBHA compounds exhibit anticancer and anti-inflammatory activities.

## 1. Introduction

Inflammation has been linked to cancer, but only in the last decade it has been possible to understand how inflammatory cells and other tumor stromal molecules stimulate tumor progression by creating a microenvironment that is enriched by the interleukin-1*β* (IL-1*β*) and interleukin-6 (IL-6) and tumor necrosis factor-*α* (TNF) cytokines, which are protagonists of chronic inflammation associated with cancers on the liver, stomach, and colon. In fact, inflammation is a condition that promotes tumor effecting on almost all types of solid cancers and then enabling many cancer features (breast, colon, and hepatocarcinoma) and promoting incipient neoplasia progression in malignant tumors complete [1]. The critical role of chronic inflammation in cancer was first proposed by Rudolf Virchow in 1863, when he observed the presence of leukocytes in neoplastic tissues [2–4].

Tumor microenvironment consists of tumor, immune, and inflammatory stromal cells, all of which produce cytokines, growth factors, and adhesion molecules that can promote tumor promotion and metastasis. It has been reported that there is an association between chronic inflammation and tumor progression and development, being related at least, and 15 of all types of cancers are attributed to inflammatory etiologies [5].

Actually, chronic inflammation acts as a regulator in tumor progression by many mechanisms, including accelerated cell proliferation, avoidance of death by apoptosis, and increase in angiogenesis and metastasis [4, 6]. Such mechanism for cancer development, in the presence of chronic inflammation, involves the continuous presence of cytokines, chemokines, ROS (reactive oxygen species), oncogenes, COX-2 (cyclooxygenase-2), 5-LOX (5-lipoxygenase), and MMPs (metalloproteinases) and activation of important transcription factors such as NF-*ĸ*B (nuclear factor-*ĸ*B), STAT-3 (signal transducer and activator of transcription 3), AP-1 (activator protein-1), and HIF-1*α* (hypoxia-inducible factor 1-alpha) [7, 8].

IL-6 is a proinflammatory cytokine associated to inflammation, which has been involved in carcinogenesis process [9, 10]. IL-6 modulates gene expression, as well as proliferation, survival, and angiogenesis, which has the presence of JAK- (Janus-kinase-) STAT signaling pathway [11]. It has been also shown that high IL-6 levels were detected in patients with systemic cancer, when compared to healthy patients or with benign diseases [12].

IL-6, similarly to TNF-*α*, facilitates tumor development, promoting the conversion of noncancerous cells in tumor stem cells. In particular, IL-6 secretion by noncancer stem cells, at poor conditions of culture adhesion, upregulates Oct4 gene expression by activation of IL-6R/JAK/STAT3 signaling pathway [13]. These findings have led researchers to propose that IL-6 is a good therapeutic target in cancer, and many clinical trials phases I/II are evaluating IL-6 or IL-6R antibodies as therapeutic alternatives [14, 15].

Another factor that was considered to be involved in inflammation and progression of many carcinomas, including colon cancer, is COX-2, an inducible form of cyclooxygenases and a limiting enzyme in the production of prostaglandins (PEGs) [16, 17]. COX-2 is progressively overexpressed during the stepwise sequence from adenoma to carcinoma and, in randomized, placebo-controlled trials, has shown that selective COX-2 inhibitors prevent recurrence of adenoma among patients with a history of adenoma or familial polyposis [18, 19].

Cancer is a serious pathology, and a substantial number of new antineoplastic agents have been discovered. Anticancer drugs that showed anti-inflammatory activity are substantially interesting, especially for the use in solid tumors, such as colon and breast cancers. The Morita-Baylis-Hillman adducts (MBHA) have been described as anticancer compounds [20–22]. It has been published that some MBHA molecules exhibited antimitotic activity against sea urchin embryonic cells [21, 22], and recently, our group demonstrated that these molecules have anticancer potential [23].

In this study, HT-29 and RAW264.7 cells were used to establish the cytotoxicity and inflammatory model in vitro, investigating the anti-inflammatory effect of MBHA compounds (A2CN, A3CN, and A4CN). Therefore, the cytotoxicity of MBHA compounds was firstly examined and anti-inflammatory activities were estimated as their inhibition against the production of NO, ROS, TNF-*α*, IL-1*β*, and IL-6 in LPS-induced RAW264.7 cells. The effects of MBHA compounds on cyclooxygenase-2 (COX-2), IL-1*β*, and IL-6 mRNA expression were also investigated in order to clarify the effect of MBHA compounds on the expression of inflammatory mediators.

## 2. Material and Methods

### 2.1. Material

A2CN (3-hydroxy-2-methylene-3-(4-nitrophenyl)-propanenitrile), A3CN (3-hydroxy-2-methylene-3-(3-nitrophenyl)-propanenitrile), and A4CN (3-hydroxy-2-methylene-3-(2-nitrophenyl)-propanenitrile) (>99% pure) were synthesized and provided as described previously [21]. A 20 mM stock solution of A2CN, A3CN, and A4CN was prepared with dimethyl sulfoxide (DMSO) and freshly diluted in culture media for all in vitro experiments and in the control condition, and cells were treated using only the vehicle. The final DMSO concentration never exceeded 0.3% (*v/v*), in either control or treated samples. Dulbecco's Modified Eagle's medium (DMEM), 3-(4,5-dimethylthiazol-2-yl)-2,5-diphenyltetrazolium bromide (MTT), lipopolysaccharide (LPS, *Escherichia coli* serotype 055 : B5), the Griess reagent, and the primers were acquired from Sigma Chemical Co. (St. Louis, Mo, USA). Fetal bovine serum (FBS) was obtained from Cripion Biotecnologia (São Paulo, SP, Brazil). Cytokine ELISA kit was purchased from eBioscience (San Diego, CA, USA). TRIzol reagent was purchased from Invitrogen (Carlsbad, CA, USA). All other chemicals used in the experiments were commercial products of reagent grade.

### 2.2. Cell Culture

Murine RAW264.7 macrophages and adenocarcinoma colorectal human HT-29 cells were purchased from the Cell Bank of Rio de Janeiro (CBRJ, Rio de Janeiro, RJ, Brazil). These cells were cultured in DMEM containing 10% FBS, penicillin (100 units/mL), and streptomycin (100 *μ*g/mL) in a 5% CO_2_-humidified incubator at 37°C. Cells were subcultured every 2 days, at a dilution of 1 : 5 using 0.05% trypsin −0.02% EDTA in Ca^2+^, Mg^2+^-free phosphate-buffered saline solution (DPBS).

### 2.3. Cell Viability-MTT Assay

The cytotoxicity of RAW264.7 and HT-29 cells was evaluated using the original enzymatic reduction of MTT assay to produce formazan crystals. Raw 264.5 cells were seeded at 1 × 10^5^ cells/well in 96-well tissue culture plate for 1 hour. Then, cells were exposed to different concentrations of A2CN, A3CN, or A4CN (2.5, 5, 10, and 20 *μ*M) dissolved in the DMEM medium used as control (CTR) with 10% FBS or being necessary, the medium was incubated with LPS (1 *μ*g/mL) in triplicate. HT-29 cells were plated overnight and treated with A2CN, A3CN, or A4CN by 24 h (5, 10, 20, 40, 80, 160, and 320 *μ*M). After 24 h of incubation, plates were centrifuged (500 ×g, 5 min) and the supernatant was removed, followed by the addition of MTT solution (0.5 mg/mL in PBS) and incubation for 4 hours at 37°C. After 4 hours, the MTT formazan product was dissolved in SDS/HCl 0.01 N and absorbance was measured at 570 nm in reader plate ELISA (BioTek ELx800, USA) [24].

### 2.4. Measurement of Nitric Oxide (NO) Production

The production of NO was determined by assaying culture supernatant for NO^2−^, a major stable product of NO. Briefly, RAW264.7 cells were plated in a 96-well plate for 1 hour. Then, cells were incubated only with the DMEM medium used as control (CTR), or with LPS (1 *μ*g/mL), or treated with A2CN, A3CN, or A4CN and added with LPS (1 *μ*g/mL) at 37°C for 22 h. After 22 h, 100 *μ*L of each supernatant was mixed with equal amount of Griess reagent (1% sulfanilamide, 0.1% N-[naphthyl]ethylenediamine dihydrochloride, and 5% phosphoric acid) at room temperature for 10 min. Absorbance of the mixture was measured at 540 nm. Nitrite concentration was calculated by comparison with a sodium nitrite standard curve [25].

### 2.5. Measurement of Intracellular Reactive Oxygen Species (ROS)

The intracellular ROS was estimated by fluorescent probe, 2′,7′-dichlorohydrofluorescein diacetate (H2-DCF-DA). This dye is deacetylated by intracellular esterase and converted to nonfluorescent 2′,7′-dichlorohydrofluorescein (H2-DCF), which is rapidly oxidized to the highly fluorescent compound 2′,7′-dichlorohydrofluorescein (DCF) in the presence of ROS. The RAW264.7 cells were dispensed into 24-well plates for 1 h. Then, the cells were incubated only with the DMEM medium used as control (CTR), or with LPS (1 *μ*g/mL), or treated with A2CN, A3CN, or A4CN and added with LPS (1 *μ*g/mL) at 37°C for 22 h. After 22 hours of exposure, the samples were removed and centrifuged (200 ×g, 5 min), washed with PBS at 37 C, and labeled with H_2_-DCFH-DA for 30 min in the dark conditions, at 37°C. Then, cells were washed with PBS and analyzed by flow cytometer in FACSCalibur (Becton Dickinson, USA) at FL1-H filter. A minimum of 10,000 events were acquired for each sample.

### 2.6. Measurement of IL-1, IL-6, and TNF Production

Cells were cultured in a 24-well plate for 24 h. Then, the cells were incubated only with the DMEM medium used as control (CTR), with LPS (1 *μ*g/mL), or treated with A2CN, A3CN, or A4CN and added with LPS (1 *μ*g/mL) at 37°C for 22 h. After treatment, RAW264.7 cell culture supernatants were collected and stored at −80°C until analysis. Cytokine production was measured with commercial mouse ELISA kits (eBioscience ELISA kits, CA, USA) following the manufacturer's protocol.

### 2.7. RNA Extraction

RAW264.7 cells were cultured in 6-well plates (1 × 10^6^ cells/mL) for 1 h and incubated only with the DMEM medium used as control (CTR), or with LPS (1 *μ*g/mL), or treated with 10 *μ*M of A2CN, A3CN, or A4CN and added with LPS (1 *μ*g/mL) for 22 h. Briefly, RNA extraction from treated or nontreated cells was performed using 1.0 mL of TRIzol for each 1 × 10^6^ cells of sample according to the manufacturer's recommendation or using RNeasy mini kit (Qiagen, CA, USA). RNA integrity was assayed by agarose gel electrophoresis and treated with DNAse (RQ1 RNAse free DNAse—Promega, USA). cDNA was performed using SuperScript III Platinum one-step qRT-PCR Systems (Invitrogen, USA).

### 2.8. Quantitative Real-Time Polymerase Chain Reaction (q-PCR) Analysis

To evaluate mRNA expression levels, total RNA was isolated from RAW264.7 cells with TRIzol reagent. Quantitative PCR was performed in a SuperScript® III Platinum® One-Step qRT-PCR kit (Invitrogen, USA), under the Bio-Rad Real-Time PCR Detection System (Bio-Rad Laboratories Inc., USA), and the results were analyzed with the CFX manager optical system software supplied with the equipment. The housekeeping gene GAPDH was used as an internal standard to quantify the levels of parameters of q-PCR reactions. Such reactions were as the following: 50°C for 2 min, 95°C for 5 min for one cycle, then 95°C for 15 s, 64°C for 30 s, and 72°C for 30 s for 50 cycles. The fluorescence signal was detected at the end of each cycle. The 2^−∆∆CT^ method was performed to analyze the results. The primers used in the experiment are shown in Table 1.

### 2.9. Statistical Analysis

The data are expressed as mean ± SEM from three replicates per treatment. Data were analyzed by one-way ANOVA followed by the Newman-Keuls analysis by multiple comparison tests. The level of significance was set at *p* < 0.05. Data of all the results in this study were obtained from at least three independent experiments with a similar pattern.

## 3. Results

### 3.1. Effect of MBHA on Cell Viability

**Figure 1:**
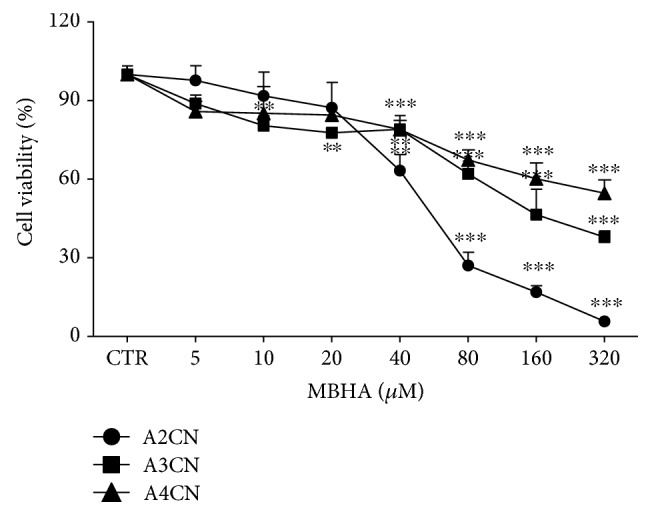
Cell viability of HT-29 cells incubated for 24 h with MBHA compounds (A2CN, A3CN, or A4CN). Data were obtained by MTT assay. CTR, cells cultured without MBHA compounds (means 0 *μ*M). Cells were treated in a medium supplemented with 10% of FBS. Results are mean ± SEM of three independent experiments performed in triplicate. ^∗∗^*p* < 0.01, ^∗∗∗^*p* < 0.001 compared with CTR. Data were analyzed by ANOVA followed by Newman-Keuls post hoc test.

**Figure 2:**
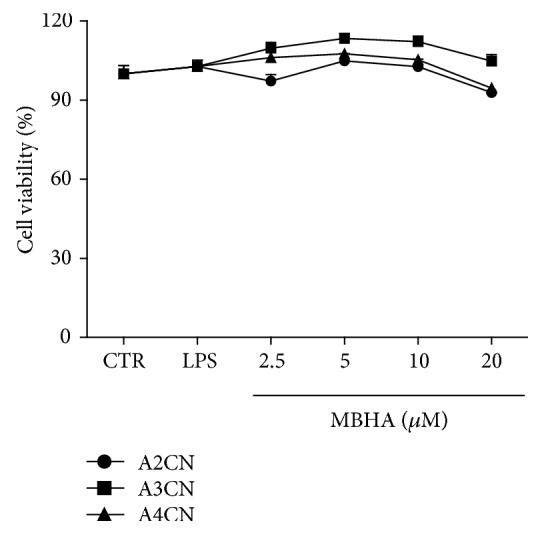
Cell viability of RAW264.7 cells incubated for 22 h with MBHA (A2CN, A3CN, or A4CN). Data were obtained by MTT assay. Cells were treated for 22 h. CTR, cells without LPS and MBHA compounds. LPS, cells incubated with LPS (1 *μ*g/mL). MBHA (*μ*M) means cells cultured with A2CN, A3CN, or A4CN as indicated. Results are mean ± SEM of three independent experiments of percentage of cell viability performed in triplicate and were analyzed by ANOVA followed by Newman-Keuls post hoc test.

We first studied the effect cytotoxic of A2CN, A3CN, and A4CN on HT-29 tumor cells (Figure 1). The molecules exhibited cytotoxicity for cells after 24 h, with CI_50_ of 54 ± 7.4, 134 ± 7.1, and 231 ± 7.5 *μ*M for A2CN, A3CN, and A4CN, respectively. However, while adducts were tested on RAW264.7 cells, these compounds did not affect the cell viability when assayed without or with incubation of 1 *μ*g/mL LPS (Figure 2).

### 3.2. Effect of MBHA on NO Production from LPS-Induced RAW264.7 Cells

**Figure 3:**
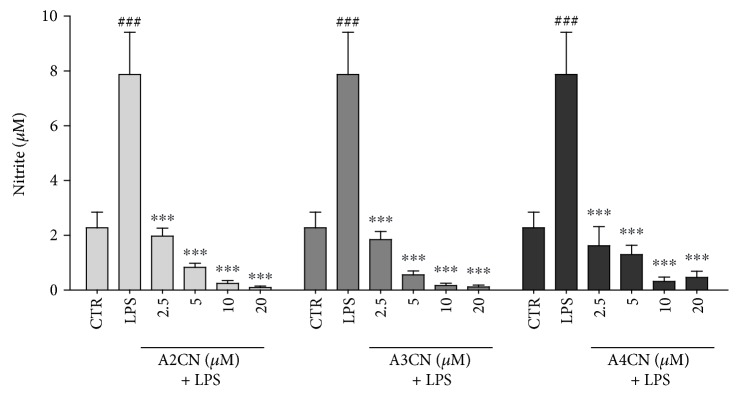
Evaluation of LPS-induced NO production incubated without compounds or coincubated with increasing concentrations of A2CN, A3CN, or A4CN for 22 h. LPS was incubated at 1 *μ*g/mL. CTR, cells without LPS and MBHA compounds. LPS, cells incubated with LPS (1 *μ*g/mL). NO production was measured using the media and Griess reagent. ^###^*p* < 0.001, compared to CTR. ^∗∗∗^*p* < 0.001, compared to LPS + MBHA compound groups. Data were analyzed by ANOVA followed by Newman-Keuls post hoc test.

Using subcytotoxic concentrations of MBHA for HT-29 cells, we tested the anti-inflammatory activity of A2CN, A3CN, and A4CN incubated with LPS (1 *μ*g/mL) on RAW264.7 cells. NO production, measured as nitrite, was increased dramatically compared with the control group. To determine the effect of MBHA on NO production, different concentrations of A2CN, A3CN, and A4CN (2.5 *μ*M, 5 *μ*M, 10 *μ*M, and 20 *μ*M incubated with LPS) were plated with the cells for 22 h. A concentration-dependent inhibition of NO generation was observed. The coincubation of the cells with LPS and A2CN, A3CN, or A4CN decreased drastically to basal concentrations (Figure 3).

### 3.3. Production of ROS by LPS-Induced RAW264.7 Cells Treated with MBHA

**Figure 4:**
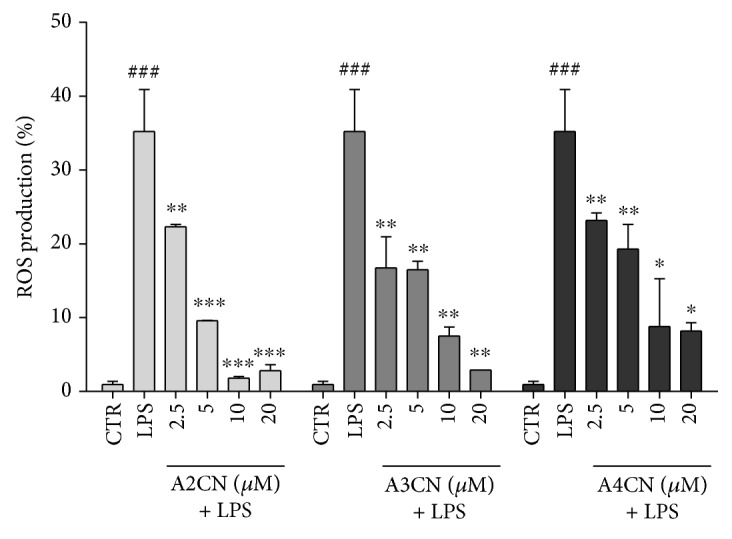
Effect of A2CN, A3CN, and A4CN on ROS production by RAW264.7 macrophage cells stimulated with LPS (1 *μ*g/mL) for 22 h of incubation. CTR means cells without LPS and MBHA compound incubation. LPS, cells incubated with LPS (1 *μ*g/mL). Cells were labeled with H2-DCFH-DA, and ROS production was quantified by flow cytometry in FL-1 channel. Results represented mean ± SEM of three experiments in triplicate. Data were analyzed by ANOVA, followed Newman-Keuls post hoc test. ^###^*p* < 0.001, compared to CTR group. ^∗^*p* < 0.05, ^∗∗^*p* < 0.01, and ^∗∗∗^*p* < 0.001, compared to LPS + MBHA compound groups.

It has been reported that mitochondrial ROS molecules act to trigger the production of inflammatory cytokines [26, 27]. Therefore, we evaluated the participation of adducts A2CN, A3CN, and A4CN in the production of ROS in RAW264.7 cells. As seen in Figure 4, all MBHA compounds decreased the ROS production induced by LPS from 2.5 *μ*M to the highest tested concentration.

### 3.4. Effect of MBHA on mRNA Expression of IL-1, IL-6, and COX-2

**Figure 5:**
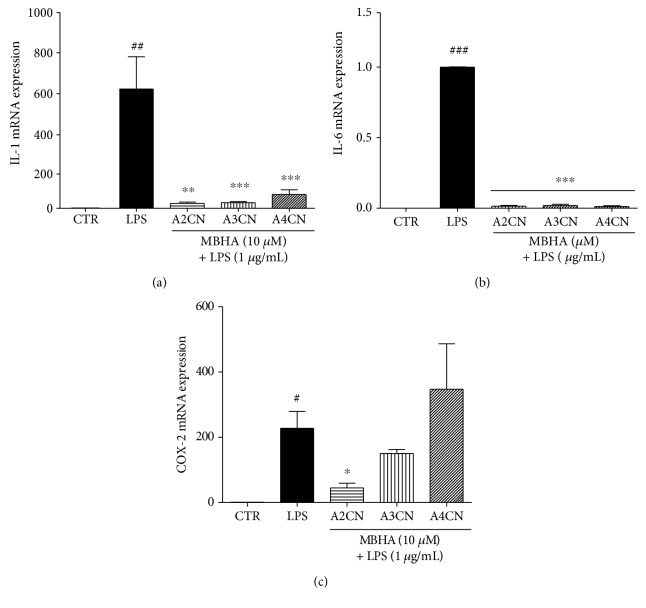
Effect on the mRNA expression of inflammatory mediators: IL-1 (a), IL-6 (b), and COX-2 (c). Cells were incubated with A2CN, A3CN, or A4CN (10 *μ*M) in the presence of LPS (1 *μ*g/mL) for 22 h. CTR means cells without LPS and MBHA compound incubation. LPS, cells incubated with LPS (1 *μ*g/mL). The mRNA levels were determined by quantitative RT-PCR using 2^−ΔΔct^ method. Results were analyzed by unpaired *t*-test. ^#^*p* < 0.05, ^##^*p* < 0.01, and ^###^*p* < 0.001, compared to CTR group and ^∗^*p* < 0.05, ^∗∗^*p* < 0.01, and ^∗∗∗^*p* < 0.001 compared to LPS + MBHA compound groups.

As shown in Figure 5, the MBHA compounds inhibited significantly the upregulation of LPS-induced mRNA expression of IL-1 (Figure 5(a)) and IL-6 (Figure 5(b)) showing almost complete suppression induced by all adducts tested. However, the inhibition of COX-2 gene occurred only in cells treated with A2CN (Figure 5(c)).

### 3.5. Effect of MBHA on IL-1, IL-6, and TNF Production from LPS-Induced RAW264.7

**Figure 6:**
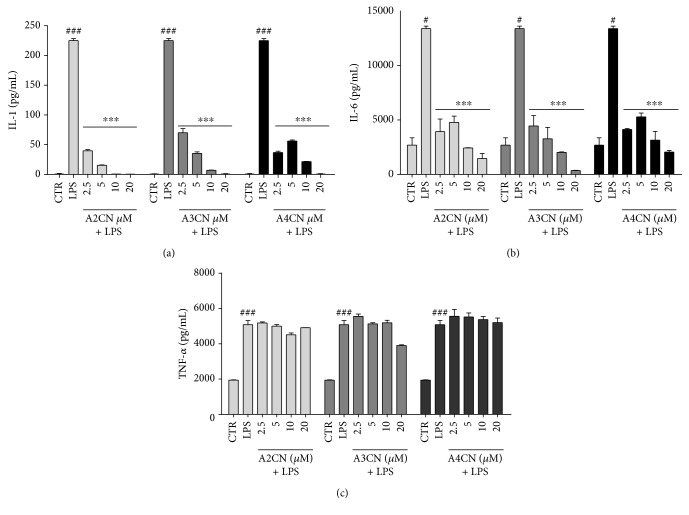
Effects of A2CN, A3CN, and A4CN compounds on inflammatory cytokine production: IL-1 (a), IL-6 (b), and TNF-*α* (c). CTR means cells without LPS and MBHA compound incubation. LPS, cells incubated with LPS (1 *μ*g/mL). Cells were incubated with A2CN, A3CN, or A4CN (2.5–20 *μ*M) in the presence of LPS (1 *μ*g/mL) for 22 h, as indicated. Reported values are the mean ± SEM of three independent experiments in triplicate. The dates were analyzed by ANOVA followed by Newman-Keuls post hoc test. ^#^*p* < 0.05, ^###^*p* < 0.001, compared to CTR group. ^∗∗∗^*p* < 0.001, compared to LPS + MBHA compound groups.

LPS is a well-known potent activator of inflammatory cytokines like NO, PGE2, TNF-*α*, IL-1, and IL-6. When RAW264.7 cells were cultured with A2CN, A3CN, or A4CN compounds for 22 h, a total inhibition of IL-1 (Figure 6(a)) and IL-6 (Figure 6(b)) productions were observed at all concentrations tested. However, TNF levels were not altered (Figure 6(c)). We also observed that incubating only an adduct compound separately, we did not verify any effect on cytokine secretion in RAW264.7 cells.

## 4. Discussion

The MBHA compounds are a class of molecules which have shown high anticancer activity against a variety of cancer cell lines [20–23]. Therefore, it is important to carry on the research and discoveries of new activities of such MBHA molecules.

Inflammation is present in cancers that have arisen without precancerous inflammation [28]. The inflammatory state is required to maintain and promote cancer progression with complete malignant phenotype, such as tissue remodeling, angiogenesis, metastasis, and suppression of innate immune response [29]. In our recent study, we demonstrated that A2CN, A3CN, and A4CN exhibited antileukemic activity [23]. In this study, we showed that A2CN, A3CN, and A4CN compounds reduced the cell viability of HT-29 cell line (Figure 1), according to previous data published by our group [23], confirming that these molecules have anticancer potential. It was reported that MBHA compounds demonstrated a poor cytotoxic effect in normal cells and these compounds have close relationship between inflammation and cancer [25]. That way, this study was designed to explore the anti-inflammatory effects of AMBH using RAW264.7 macrophage cells. Actually, discoveries of molecules which are cytotoxic to tumor cells and inhibit inflammatory process have great importance at advancement of therapies for solid tumors.

Initially, we examined the effects of MBHA compounds on cell viability in RAW264.7 macrophage cells. The MTT assay showed that treatment of A2CN, A3CN, and A4CN at concentrations of 2.5–20 *μ*M did not exhibit any cytotoxic effect in RAW264.7 cells (Figure 2). Based on the results above, we selected these noncytotoxic concentrations for such MBHA compounds, which were further examined for their anti-inflammatory properties.

One of the most prominent phenomena observed in inflammation event is the progressive increase of NO. This radical is involved in inflammation-induced human diseases such as cancer, rheumatoid arthritis, diabetes, septic shock, and cardiovascular diseases [17]. In this work, A2CN, A3CN, and A4CN compounds completely inhibited NO production in RAW264.7 cells stimulated with LPS, even at low concentrations, 2.5 *μ*M. These results suggest that MBHA compounds can exert an anti-inflammatory effect. The NO inhibition did not change the cell viability according to the data obtained after 22 h treatment with 20 *μ*M MBHA compounds (Figure 2).

Chronic inflammation is often accompanied by increased production of reactive oxygen species (ROS). The proneoplastic activity of ROS can induce DNA damage [19]. Agents that prevent the formation of ROS can also inhibit the induction of DNA damage, mutagenesis, and cell transformation. The adducts A2CN, A3CN, and A4CN incubated with LPS almost completely inhibited ROS production in RAW264.7 cells, even at low concentration experienced, and this result together with the reduced synthesis of NO provoked by such compounds, clearly demonstrate the antiredox potential of MBHA molecules. Additionally, we also showed that MBHA compounds in a dose-dependent manner reduced LPS-induced ROS production in RAW264.7 cells. In fact, ROS, including hydrogen peroxide and superoxide anions, are potent inducers of various signaling pathways encompassing MAPKs and JAK-STAT pathways [30].

For a more clear understanding of A2CN, A3CN, and A4CN effects, we evaluated whether the change in the levels of mRNA for the genes IL-1*β* and IL-6 in RAW264.7 cells stimulated with LPS and treated with 10 *μ*M of all MBHA might occur. These compounds inhibited the gene expression of IL-1*β* and IL-6 to basal levels (incubations without LPS stimulation). On the other hand, these genes were detected only when the cells were stimulated with LPS (Figures 5(a) and 5(b)). Numerous studies have indicated that tumor cells exhibit constitutive production of TNF-*α* proinflammatory cytokines, IL-1*α*, IL-6, and GM-CSF (macrophage-granulocyte colony-stimulating factor) [4, 27]. In this study, inhibition of IL-1 and IL-6 genes in inflammatory cells suggests that blocking can also happen in tumor cells, which produce these cytokines, favoring the tumor progression.

COX-2 is an inducible enzyme and can be affected by mitogens, growth factors, and hormones, which are of great importance in tumorigenesis. COX-2 can also induce VEGF production, contributing to angiogenesis, as well as increases metalloproteinases, which improve the invasion of tumor vessels and reduce the production of antiangiogenic cytokines, such as IL-12. It has also been shown that COX-2 increases the resistance to apoptosis [31]. COX-2 selective inhibitors are better tolerated at therapeutic doses, not inhibiting COX-1. These COX-2 selective inhibitors have helped to suppress tumor growth and malignant transformation, by stimulating apoptosis, and inhibit the VEGF production by reducing angiogenesis [31, 32]. In this work, we demonstrated that only A2CN at a concentration of 10 *μ*M was able to decrease the expression of COX-2 in RAW264.7 cells stimulated with LPS (Figure 5). In this case, position of the nitro group isomer influenced the molecule effectiveness to inhibit the enzyme gene expression.

Once it has recently demonstrated the action of mitochondrial ROS (MTROS) as signaling molecules to trigger the production of proinflammatory cytokines such as IL-6 and TNF-*α* [30], we sought to demonstrate the action of MBHA in the production of these inflammatory cytokines in RAW264.7 cells stimulated with LPS. The data obtained by ELISA showed that cytokine production by RAW264.7 cells after stimulation with LPS was greatly altered. The release of IL-1*β* and IL-6 was completely blocked after 22 h by addition of A2CN, A3CN, or A4CN, even at the lowest concentration of 2.5 *μ*M (*p* < 0.001). However, the cytokine TNF-*α* was not affected by any of the tested molecules. This result demonstrates a potent anti-inflammatory effect in vitro in subcytotoxic concentrations. It is suggested that the inhibition of IL-6 production is mediated by the inhibition of IL-1*β*.

Direct evidence has shown that IL1-*β* plays an important role in multiple myeloma, and when released, this cytokine induces IL-6 production by bone marrow stromal cells and acts as an autocrine growth factor for myeloma cells [4, 33]. IL1-*β* also regulates HIF-1*α* protein, involving an inflammatory signaling pathway to NF-*ĸ*B and COX-2, resulting in the upregulation of VEGF (vascular endothelial growth factor), a potent angiogenic factor needed for metastasis and for tumor growth [34]. IL-6 is another proinflammatory cytokine that has been involved in carcinogenesis associated with inflammation [9, 10]. This cytokine modulates the expression of genes involved in the proliferation, survival, and angiogenesis by signaling via the Janus-kinase- (JAK-) STAT [11].

As far as we know, the data shown on this paper shows for the first time an anti-inflammatory activity of A2CN, A3CN, and A4CN compounds, which especially blocks the IL-1 and IL-6 cytokines and consequently may reduce the progression and growth of tumors.

In addition, we showed that A2CN compound proved to be the most effective molecule in the anti-inflammatory action among the tested isomers because this adduct was also able to inhibit the expression of COX-2 gene. This considerable A2CN activity is very promising because it contributes to the anticancer activity, recently demonstrated by our group in several cancer cell lines [23], and corroborates the potent antiparasitic activity against Leishmania, Plasmodium and, Trypanosoma, widely reported in the literature.

Most antineoplastic actions with nitro (nitacrine, 1-(1,5-dicloropentano-3-yl)-4-nitrobenzene) and antiparasitic (metronidazole, tinidazole, secnidazole, benznidazole, and nifurtimox) are dependent upon the enzyme bioreduction process group nitro, strong electron acceptor, as probable mechanism of action. The passage of these molecules generally occurs by passive diffusion in the membrane, and the bioreduction process generates a lot of free radicals' short life, resulting in peroxidation of biological and protein membranes, causing damage to the DNA [35]. Nimesulide is an example of nitro compound, nitrobenzene's class of derivatives, used in anti-inflammatory therapy, and extensive clinical application. Thus, the nitro group present in molecules tested in this study and their position contribute to their cytotoxic activity on tumor cells, as well as for its anti-inflammatory activity.

The increase production of free radicals occurs due to decreased activity of cellular defense systems. The balance in macrophage stimulated with LPS is disrupted by excessive production of ROS in a mutual induction pattern. In this study, we also observed that A2CN, A3CN, and A4CN dose dependently reduced LPS-induced ROS production in RAW264.7 cells. In fact, ROS, including hydrogen peroxide and superoxide anions, are potent inducers of various signaling pathways encompassing MAPKs and JAK-STAT pathways [36]. Further work is necessary to investigate the precise correlation between ROS and JAK/STAT signaling.

## Figures and Tables

**Table 1:** Primer sequences used.

Gene	Primer sequence	Product size	NM code
IL-6	F: 5′-GGGACTGATGCTGGTGACAA-3′ R: 5′-TAACGCACTAGGTTTGCCGA-3′	599 pb	NM_031168.1
IL-1*β*	F: 5′-AACCTTTGACCTGGGCTGTC-3′ R: 5′-AATGGGAACGTCACACACCA-3′	253 pb	NM_008361.3
COX-2	F: 5′-CGTAGCAGATGACTGCCCAA-3′ R: 5′-TCTCAGGGATGTGAGGAGGG-3′	383 pb	NM_011198.3
GAPDH-c	F: 5′-GACCACAGTCCATGCCATCA-3′ R: 5′-TAGGGCCTCTCTTGCTCAGT-3′	535 pb	NM_0080084.2
